# Molaren-Inzisiven-Hypomineralisation (MIH). Häufigkeit und mögliche Ursachen unter besonderer Berücksichtigung der Ergebnisse aus den Münchner Geburtskohorten GINIplus und LISA

**DOI:** 10.1007/s00103-021-03366-1

**Published:** 2021-07-02

**Authors:** Jan Kühnisch, Marie Standl, Reinhard Hickel, Joachim Heinrich

**Affiliations:** 1 Poliklinik für Zahnerhaltung und Parodontologie, Klinikum der Universität München, Ludwig-Maximilians-Universität München, München, Deutschland; 2 Institut für Epidemiologie, Helmholtz Zentrum München – Deutsches Forschungszentrum für Gesundheit und Umwelt, Neuherberg, Deutschland; 3 Institut und Poliklinik für Arbeits‑, Sozial- und Umweltmedizin, Klinikum der Universität München, Ludwig-Maximilians-Universität München, München, Deutschland

**Keywords:** Strukturstörung der Zähne, Entwicklungsbedingte Schmelzdefekte, Opazitäten, Atypische Restaurationen, Epidemiologie, Ätiologie, Dental developmental defects, Demarcated opacities, Enamel breakdowns, Atypical restoration, Epidemiology, Etiology

## Abstract

Die Molaren-Inzisiven-Hypomineralisation (MIH) – mittlerweile auch bekannt unter dem Begriff der Kreidezähne – stellt heute neben der Karies eine häufige Erkrankung der Zähne im Kindes- und Jugendalter dar. Neben den ästhetischen Einschränkungen insbesondere an den Frontzähnen sind Hypersensibilitäten und Schmelzeinbrüche an bleibenden Molaren für die Betroffenen von funktioneller Bedeutung. Während die Häufigkeit der MIH in einer Größenordnung zwischen ~ 10 % und ~ 30 % liegt und gut beschrieben ist, stellt sich die Situation bezüglich der Ursachenforschung unbefriedigend dar. Obwohl in der Vergangenheit Anstrengungen zur Klärung der Ätiologie unternommen wurden, liegt bis in die Gegenwart keine plausible Ätiologiekette vor. Ursachenforschungen sind dabei als methodisch anspruchsvoll zu beurteilen, da diese optimalerweise in prospektiv geplante Geburtskohortenstudien eingebettet sein sollten, welche spätestens mit der Geburt beginnen. Ziel des vorliegenden Beitrages ist es, die klinische Charakteristik der MIH, Häufigkeiten und potenzielle Ursachen unter besonderer Berücksichtigung bereits publizierter Ergebnisse aus den beiden Münchner Geburtskohortenstudien GINIplus und LISA zusammenfassend darzustellen.

## Abstract (en)

After caries, molar incisor hypomineralisation (MIH)—also known as chalky teeth—is one the most common dental diseases in children and adolescents. In addition to aesthetic limitations, especially on the anterior teeth, hypersensitivities and enamel breakdowns on permanent molars are of functional importance. While the MIH prevalence rates range from ~ 10% to ~ 30% and is well described, the situation regarding aetiology is unsatisfactory. Although efforts have been made in the past to clarify the aetiology, no plausible reason is available so far. Aetiology research has to be judged as methodologically challenging, since it should ideally be embedded in prospectively planned birth cohort studies. The aim of this article is to summarize typical clinical characteristics of MIH, epidemiological findings and potential causes with special reference to the earlier published results from the two Munich birth cohort studies GINIplus and LISA.

## Definition und Diagnostik der MIH

Die Molaren-Inzisiven-Hypomineralisation (MIH) stellt eine entwicklungsbedingte Störung der Zahnschmelzbildung dar, welche durch eine oder mehrere nach wie vor unbekannte, systemisch wirkende Noxen bedingt wird [1–3]. Der oder die verursachende/n Faktor/en oder Substanz/en führen zu einer Störung der regulären Schmelzbildung durch die Ameloblasten und in der Folge sind sowohl Hypomineralisationen – als qualitative Normabweichungen – sowie Hypoplasien – als quantitative Schmelzdefekte – beobachtbar. Die Störung kann prinzipiell an allen Milch- und bleibenden Zähnen auftreten, wobei die bleibenden Schneidezähne (Inzisiven) und ersten bleibenden Backenzähne (Molaren) die am häufigsten betroffenen Zahngruppen darstellen [4, 5]. Da die betroffenen Zahngruppen in der frühen Kindheitsphase mineralisieren, müssen die Ursachen in dieser Altersphase gesucht werden. Erheblich erschwert wird die Ursachenfindung durch die Tatsache, dass hypomineralisierte und/oder hypoplastische Zähne erst nach dem 6. Lebensjahr in die Mundhöhle durchbrechen und dann als erkrankt erkannt werden können.

**Figure 1:**
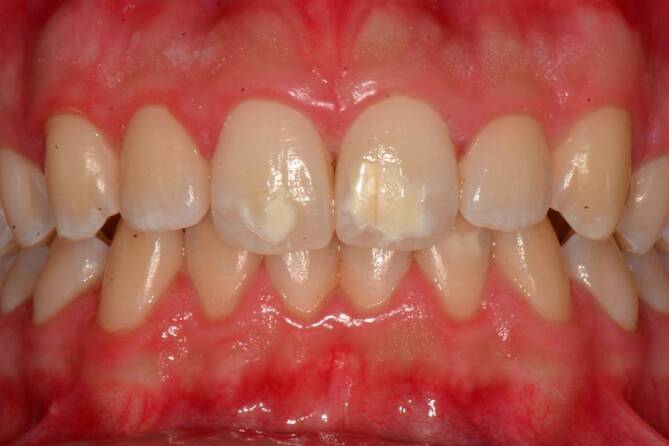

**Figure 2:**
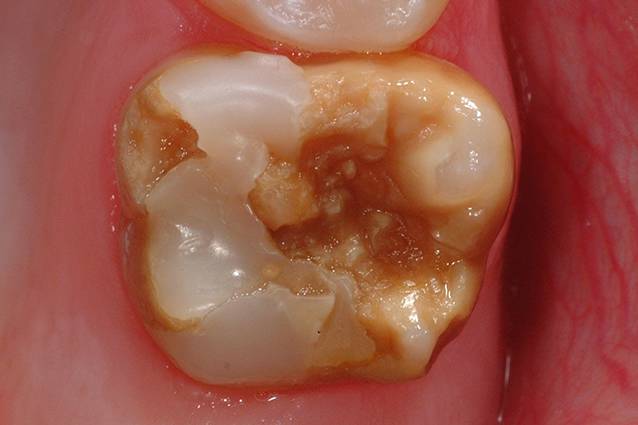

Das zur Diagnose einer MIH führende Leitsymptom sind scharf begrenzte Hypomineralisationen bzw. Opazitäten von weißer, gelblicher oder brauner Farbe im Zahnschmelz (engl. „demarcated opacities“, Abb. 1). Diese können darüber hinaus mit prä- und/oder posteruptiven Schmelzeinbrüchen (engl. „enamel breakdowns“ oder „enamel desintegration“) vergesellschaftet sein (Abb. 2). Zähne mit präeruptiven Defekten brechen mit einem partiell fehlenden Zahnschmelz in die Mundhöhle durch. Posteruptive Defekte treten demgegenüber erst im Laufe der kaufunktionellen Belastung auf. Die Okklusal- bzw. Kauflächen der ersten bleibenden Molaren gelten hier als prädisponierte Lokalisation. Diese Oberflächendefekte bedürfen oftmals einer restaurativen Therapie und liegen außerhalb der kariestypischen Lokalisationen. Daher hat sich für MIH-bedingte Restaurationen der Begriff der „atypischen Restaurationen“ etabliert [2].

Neben den visuell gut beurteilbaren klinischen Zeichen stellen Überempfindlichkeiten (Hypersensibilitäten, engl. „hypersensitivities“) mit unterschiedlichen Ausprägungsgraden auf thermische, chemische oder mechanische Reize ein wiederkehrendes Begleitsymptom dar. Insbesondere der Symptomenkomplex aus Schmelzeinbrüchen und Hypersensibilitäten kann zu Einschränkungen der Funktionalität, Mundhygiene und Behandlungsfähigkeit betroffener Zähne führen. Diese Faktoren gelten in der klinischen Praxis zudem als empirische Prädiktoren für kooperationsbedingte Einschränkungen oder eine Zahnarztangst [6].

Prinzipiell kann der Kliniker MIH-typische Befunde an allen Zähnen der primären bzw. bleibenden Dentition diagnostizieren. Dabei legt das klinische Erscheinungsbild nahe, dass es sich um die gleiche Entität handelt. Dieser Annahme steht jedoch in gewisser Weise eine unterschiedliche Namensgebung für das Milch- und bleibende Gebiss gegenüber. Während der Begriff „MIH“ ausschließlich für das bleibende Gebiss gilt, indem die ersten bleibenden Molaren als Indexzähne zur Diagnosestellung herangezogen werden [2], verweisen die Diagnosenamen „deciduous molar hypomineralisation“ oder „second primary molar hypomineralisation“ (dt.: Milchmolaren-Hypomineralisation) auf davon abweichende Indexzähne.

Differenzialdiagnostisch sind scharf begrenzte Hypomineralisationen von Karies, Fluorosen und anderen Strukturstörungen abzugrenzen. Unter Verweis auf die Unterschiede zwischen erworbenen und entwicklungsbedingten Defekten der Zahnhartsubstanz sind vor allem der Zeitpunkt des Auftretens sowie die Lokalisation von Bedeutung. Entwicklungsbedingte Störungen der Zahnhartsubstanz, u. a. MIH oder Fluorose, werden grundsätzlich während der Zahnentwicklung determiniert und brechen somit bereits strukturverändert in die Mundhöhle durch. Demgegenüber entsteht Karies erst nach dem Zahndurchbruch und typischerweise an den Prädilektionsstellen der Fissuren, Grübchen oder Approximalflächen (Flächen am Zahnzwischenraum). Diese Lokalisationen sind konträr zu denen, an welchen eine MIH detektierbar ist, z. B. inzisale Kronenhälften oder Höcker. Der wesentliche Unterschied zwischen MIH und Fluorose besteht darin, dass Letztere durch unscharfe Hypomineralisationen des Zahnschmelzes charakterisiert ist [7, 8]. Auch sind fluorotische Schmelzveränderungen in der Regel generalisiert und damit symmetrisch anzutreffen.

## Die Ätiologie der MIH ist unbekannt

Wie bereits in der Einleitung ausgeführt gelten die Ursachen der MIH als weitgehend ungeklärt. Diese unbefriedigende Situation verhindert letztlich eine kausal ausgerichtete Präventionsstrategie und begründet die Bedeutung entsprechender Anstrengungen in der Ätiologieforschung. Unter Verweis auf den mehrjährigen Verzug zwischen dem Zeitpunkt der etwaigen Initiation der Strukturstörung in der Säuglings- oder Kleinkindphase und dem Zeitpunkt des Zahndurchbruchs jenseits des 6. bis 8. Lebensjahres wird deutlich, dass es im Idealfall prospektiv angelegter, longitudinaler Beobachtungsstudien bedarf, welche spätestens mit der Geburt des Kindes ihren Ausgangspunkt nehmen sollten.

## Bedeutung der Geburtskohorten GINIplus und LISA

Insgesamt gibt es weltweit nur wenige prospektiv angelegte Geburtskohortenstudien, welche eine zahnmedizinische Untersuchung mit dem Ziel der Erfassung der MIH inkludierten. Dazu zählen die Generation-R-Studie^1^ in den Niederlanden, die COPSAC-Studie^2^ in Dänemark, die LIFE-Child-Studie^3^ in Leipzig sowie die beiden Münchner Kohorten GINIplus^4^ und LISA^5^. Allen Kohorten ist gemeinsam, dass Kinder spätestens mit der Geburt in das Untersuchungsprogramm aufgenommen wurden und von diesem Zeitpunkt an kontinuierlich anthropometrische, medizinische, soziale und Lifestyleinformationen gesammelt wurden. Als nachteilig ist aus heutiger Sicht zu beurteilen, dass die Aufnahme zahnmedizinischer Untersuchungsmodule bei allen Kohorten i. d. R. erst nach der Studieninitiation erfolgte. Dies führte dazu, dass potenziell relevante Variablen für die Entstehung einer MIH in der perinatalen Phase oder in den ersten Lebensjahren mitunter nicht oder nur unvollständig prospektiv dokumentiert werden konnten. Trotz bestehender Datenlücken sind die gesammelten Daten als Glücksfall für die MIH-Forschung zu beurteilen, da es damit erstmals gelang, Daten aus der frühen Kindheit valide mit dem erst Jahre später diagnostizierbaren Auftreten von MIH-Zähnen zu verknüpfen.

## Methodik von GINIplus- und LISA

Das longitudinale Studiendesign, die Zielstellungen, Ein- und Ausschlusskriterien zur Rekrutierung, Follow-up-Untersuchungen sowie die jeweiligen Untersuchungsmodule der GINIplus- und LISA-Kohorten sind ausführlich beschrieben [9]. Die zahnärztlichen Untersuchungen fanden im Rahmen der 10- und 15-Jahres-Follow-ups statt. Im Anschluss an die jeweiligen ärztlichen Untersuchungen wurde unter Feldbedingungen der Zahnstatus durch trainierte und kalibrierte Zahnärzte mit stumpfer Sonde, zahnärztlichem Spiegel und bei guter Ausleuchtung der Mundhöhle erhoben. Alle Teilnehmer wurden vor der Untersuchung gebeten ihre Zähne zu reinigen. Als Parameter der Mund- und Zahngesundheit wurden der Gingivitisstatus, der Kariesstatus als DMF-Index (Kariesindex) unter Einschluss nicht kavitierter kariöser Läsionen und das Vorhandensein von Molaren-Inzisiven-Hypomineralisationen ([1, 2]; Abb. 1 und 2) erhoben. Die Erhebung der Zahnhartsubstanzdefekte erfolgte standardisiert an allen vorhandenen Zähnen bzw. Zahnflächen, d. h. bei 10-Jährigen zu Beginn der zweiten Wechselgebissphase (*N* = 1158) und bei 15-Jährigen in der vollständigen bleibenden Dentition (*N* = 1302). Mit diesem Vorgehen war es im Rahmen der statistischen Auswertungen dann möglich, eine Phänotypisierung auch unabhängig von einzelnen Indexzähnen vorzunehmen. Dieser methodische Aspekt ist insofern von Bedeutung, als dass die Diagnose einer MIH immer noch davon abhängig gemacht wird, ob zumindest ein erster bleibender Molar von einer Hypomineralisation betroffen ist [2]. Dies sollte aus Sicht der Autoren durchaus offener diskutiert werden [3, 10].

## Häufigkeit und klinische Ausprägung der MIH

Die in den vergangenen Jahrzehnten initiierten epidemiologischen Erhebungen dokumentierten im internationalen Maßstab Prävalenzraten von MIH zwischen ~ 10 % und ~ 30 % für Kinder und Jugendliche [4, 11, 12], sofern die ersten bleibenden Molaren als Indexzähne herangezogen werden. Epidemiologische Daten für den deutschsprachigen Raum liegen in etwa in der gleichen Größenordnung. Insgesamt wiesen 31,6 % der 10-Jährigen und 40,2 % der 15-Jährigen mindestens eine Hypomineralisation in der bleibenden Dentition und unabhängig von Indexzähnen in den beiden Münchner Kohorten auf. Diese Größenordnung fand sich mit 28,7 % auch in der fünften deutschen Mundgesundheitsstudie [13].

Die Prävalenz der MIH entsprechend der Definition der European Academy of Paediatric Dentistry (EAPD) – mindestens ein erster bleibender Molar muss betroffen sein [2] – lag bei den untersuchten 10- und 15-Jährigen in München bei 13,6 % bzw. 17,2 % [5] und befindet sich damit in etwa gleichen Größenordnungen wie in den Großstädten Hamburg mit 14,0 % oder Düsseldorf mit 14,6 % [14]. Die für andere deutschsprachige Regionen verfügbaren Häufigkeitsraten lagen niedriger: 5,6 % bei 10- bis 17-Jährigen in Dresden [15], 5,9 % bei 6‑ bis 12-Jährigen in Gießen [16], 10,9 % bei > 7-Jährigen in Salzburg bzw. Tirol [17], 4,3 % in Greifswald und 6,0 % in Heidelberg bei 7‑ bis 10-Jährigen [14]. Zu erwähnen ist, dass methodische Abweichungen zwischen den einzelnen Studien auch zu unterschiedlichen Ergebnissen führen können. Letztlich bleibt aber zu konstatieren, dass die MIH ein prävalentes zahnärztliches Erkrankungsbild darstellt.

**Figure 3:**
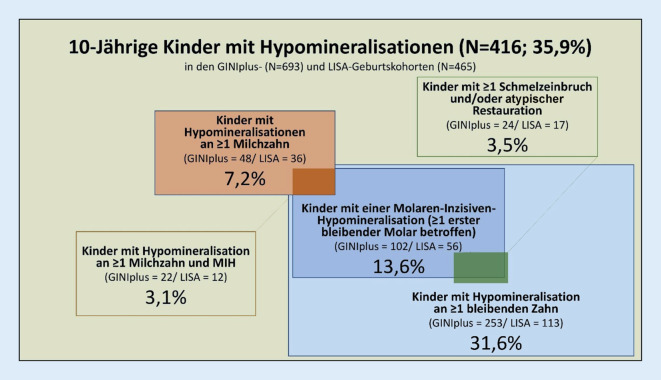

Vergleichsweise selten wurde das gemeinsame Auftreten von Hypomineralisationen an Milch- und bleibenden Zähnen im Detail betrachtet. In den Münchner Kohorten fanden sich bei 7,2 % der 10-Jährigen Hypomineralisationen in der Milchzahndentition bei einer oben schon erwähnten Häufigkeit der MIH von 13,6 % (Abb. 3). Werden die Kinder mit Hypomineralisationen an mindestens einem Milchzahn (*N* = 84) und einer MIH (*N* = 158) als Bezugsgröße gewählt, so fanden sich Hypomineralisationen in beiden Dentitionen lediglich bei 34 Individuen (14,0 %). Dies legt die Schlussfolgerung nahe, dass die Präsenz von Hypomineralisationen an Milchzähnen kein sicherer Prädiktor für das Auftreten einer MIH in der bleibenden Dentition ist.

Werden die verfügbaren, zahnbezogenen Analysen zur Verteilung der MIH betrachtet, so wurde offensichtlich, dass mehr als 90 % aller MIH-Zähne lediglich abgegrenzte Opazitäten ohne Oberflächendefekte aufweisen. Damit ist zu postulieren, dass die Mehrzahl der Betroffenen lediglich moderat ausgeprägte funktionelle und/oder ästhetische Einschränkungen aufweisen.

In verschiedenen Studien zeigten nur 5,9 % [4], 7,0 % [5] oder 5,3 % [13] aller MIH-Zähne Zahnhartsubstanzdefekte in Form von unversorgten Schmelzeinbrüchen bzw. atypischen Restaurationen. In der Gruppe der Münchner 10-Jährigen lag der Anteil der Kinder, welche restaurative Bedarfe aufwiesen bzw. bereits MIH-bedingte Restaurationen erhalten hatten, bei 3,5 % bezogen auf die Gesamtpopulation (Abb. 3). Daher kann einerseits argumentiert werden, dass restaurative Bedarfe bei MIH-Kindern nicht den Regelfall repräsentieren. Andererseits darf auch nicht außer Acht gelassen werden, dass gerade diese Kinder aufgrund von umfangreichen Zahnhartsubstanzdefekten, schmerzhaften Hypersensitivitäten, wiederholten Therapiebedarfen und/oder Kooperationseinschränkungen eine besondere Herausforderung in der täglichen Praxis des Zahnarztes darstellen [18–22].

Häufig wird ein möglicher Zusammenhang zwischen dem Auftreten von MIH und Karies diskutiert. Während eine Übersichtsarbeit [23] diese Annahme grundsätzlich untermauert, signalisieren die Daten aus den Münchner Geburtskohorten ein leicht abweichendes Bild. Bei den 10-Jährigen konnte zwar kein signifikant erhöhter Kariesbefall bei MIH-Kindern nachgewiesen werden [24]. In der Gruppe der 15-Jährigen wurde jedoch bei den MIH-Betroffenen ein DMFT von 1,1 und bei Nichtbetroffenen von 0,9 registriert [5]. Obwohl der Unterschied als signifikant ausgewiesen wurde, ist die Differenz als geringfügig zu beurteilen und sollte demzufolge keine Überbewertung erfahren.

## Mögliche Ursachen der MIH

Bezüglich der Ursachenforschung besteht ein Konsens, dass eine Beeinträchtigung oder Schädigung der Ameloblasten während der Phase der Schmelzentwicklung vorliegen muss, damit eine MIH entstehen kann. Das dafür notwendige Zeitfenster reicht vom Ende der Schwangerschaft bis in die ersten Lebensjahre hinein, da in dieser Lebensphase die typischerweise betroffenen Zähne – Milchmolaren, erste bleibende Molaren und bleibende Inzisiven – angelegt, ausgebildet und mineralisiert werden. In der Literatur wurde eine Vielzahl an Faktoren vorgeschlagen, untersucht oder diskutiert, die potenziell eine MIH auslösen könnten. Der Blick in die verfügbaren, systematischen Übersichtsarbeiten offenbart jedoch, dass die konkreten Ursachen nach wie vor als unbekannt anzusehen sind [25–28]. Von grundsätzlicher Bedeutung scheint dabei die Beobachtung zu sein, dass es sich bei der MIH mit einer gewissen Wahrscheinlichkeit um ein neuzeitliches Problem handelt [29, 30]. Die folgenden Ausführungen sollen dazu dienen, die hauptsächlich diskutierten Ursachenkomplexe zusammenzufassen.

### Erkrankungen in der Kindheit

Als eine wesentliche Ursachengruppe sind frühkindliche Infekte bzw. Kindererkrankungen zu diskutieren. Hierbei wurden wiederholt Fieber oder respiratorische Erkrankungen mit dem Auftreten einer MIH in Verbindung gebracht [27, 31]. Im Rahmen der eigenen Analysen in den GINIplus- und LISA-Kohorten blieben respiratorische Erkrankungen im Wesentlichen der einzige signifikante Faktor, der mit MIH assoziiert war [32]. Kinder der GINIplus-Kohorte, welche zumindest einmal an Asthma bronchiale, Bronchitis, Lungenentzündung oder Pseudokrupp in den ersten 4 Lebensjahren gelitten hatten, wiesen eine signifikant erhöhte Wahrscheinlichkeit auf, eine ausgeprägte MIH zu entwickeln. Eine weiterführende Exploration der Daten unter Einbeziehung von Medikamenten oder Antibiotika war nicht möglich, da diese Informationen aus der frühkindlichen Lebensphase nur lückenhaft verfügbar waren. In einer weiteren Analyse wurde kein Zusammenhang zwischen dem Auftreten von Symptomen der Aufmerksamkeitsdefizit- und Hyperaktivitätsstörungen oder Asthma und MIH beobachtet [33, 34].

### Ernährung

Der Einfluss der Säuglingsernährung auf das Auftreten von MIH wurde erstmals in den 1990er-Jahren untersucht. Hier verwiesen Alaluusua et al. [35, 36] auf einen möglichen Zusammenhang zwischen dem Stillen und dem Auftreten einer MIH. Die Autoren nahmen an, dass Umwelttoxine über die Muttermilch in den kindlichen Körper gelangen und potenziell ameloblastenschädigend wirken können. In einer späteren Untersuchung der finnischen Forschergruppe konnten die initialen Ergebnisse jedoch nicht bestätigt werden [37]. Diese Ergebnisse finden sich in Übereinstimmung mit Daten aus den Münchner Geburtskohorten. Hier konnten ebenfalls keine Assoziationen zwischen Stillen und MIH gefunden werden [32].

### Medikamente

Neben den o. g. Erkrankungen spielt deren medikamentöse Therapie bei der MIH-Initiation möglicherweise eine Rolle. Mit Blick auf die Bedeutung von infektionsbedingten Erkrankungen im Kindesalter soll an dieser Stelle vordergründig die systemische Antibiotikaeinnahme thematisiert werden. Diese kann im frühen Kindesalter potenziell ameloblastenschädigend wirken. Sie ist seit gut 50 Jahren auch im Kindesalter im klinischen Einsatz und könnte daher als Ursache für MIH infrage kommen. Studien haben den Antibiotikaeinfluss sowohl im Tierversuch [38] als auch in kindlichen Kohortenstudien [38, 39] dokumentiert. Im Tierversuch interferieren Antibiotika mit dem Ameloblastenstoffwechsel, sodass ein beschleunigtes Wachstum der Schmelzprismen eintritt, ohne dass parallel dazu eine regelrechte Mineralisation der Zahnhartsubstanz vonstattengeht [38]. Bei den Betrachtungen zu einem möglichen Einfluss von Asthma [34] konnte zudem die Bedeutung der Asthmamedikation geprüft werden. Bei Asthmatikern, die auf den Gebrauch von Asthmasprays angewiesen waren, zeigte sich kein signifikanter Zusammenhang mit dem Auftreten einer MIH. Umgekehrt wurde eine schwache Signifikanz hinsichtlich des Vorkommens von MIH bei Asthmatikern dokumentiert, die keine Asthmasprays nutzten [34].

### Vitamin D

Zusammenhänge zwischen dem Vitamin-D-Stoffwechsel und dem Auftreten einer MIH wurden mehrfach untersucht. Während die Ergebnisse zum Zusammenhang zwischen dem Serum-Vitamin-D-Spiegel und dem klinischen Auftreten einer MIH als heterogen einzustufen sind [40, 41], zeigten jüngst publizierte Daten aus der COPSAC-Studie, dass eine vorgeburtliche, hochdosierte Vitamin-D-Gabe mit einer signifikant niedrigeren Häufigkeitsrate von MIH im Alter von 6 Jahren einherging [42]. Dies deutet auf einen möglichen präventiven Effekt der Vitamin-D-Gabe in dieser Entwicklungsphase hin.

### Umweltfaktoren

Seit Beginn der Suche nach den Ursachen der MIH wird über den Einfluss von Umweltfaktoren diskutiert. Ein umwelttoxikologischer Einfluss wird bereits seit den 1990er-Jahren für Dioxine oder Bisphenol‑A (BPA) diskutiert, welche potenziell über die Muttermilch aufgenommen werden [35, 36]. Jüngste tierexperimentelle Untersuchungen zeigten für Ratten und deren BPA-Exposition strukturelle Veränderungen in der Zahnhartsubstanz auf, welche von den Autoren als MIH interpretiert wurden [43]. Unabhängig davon erscheinen weitere Untersuchungen erforderlich, um mögliche Expositionswege und kritische Aufnahmemengen für kindliche Körper abschätzen zu können.

### Genetik

In der jüngsten Vergangenheit wurde ein möglicher genetischer Einfluss für das Auftreten einer MIH verstärkt diskutiert und durch erste Untersuchungen untermauert (u. a. [3, 44, 45]). Während eigene GWAS-Analysen (Genome-wide Association Study) diese Ergebnisse bisher nicht bestätigen konnten [46], sollten zukünftige Studienprojekte genetische Untersuchungen mit inkludieren und auch dementsprechende Fallzahlen aufweisen.

## Schlussfolgerungen

Unter Verweis auf die epidemiologische Datenlage bleibt zusammenfassend zu schlussfolgern, dass es sich bei der MIH um eine prävalente Strukturstörung der Zähne handelt. Diese geht für die Betroffenen mit funktionellen und ästhetischen Beeinträchtigungen einher. Dabei sind die bleibenden Zähne deutlich häufiger als die Milchzähne betroffen. Da in den vergangenen Jahren keine alleinige Ursache für das Auftreten einer MIH identifiziert und umgekehrt jedoch verschiedene Variablen als signifikant erkannt werden konnten, ist aktuell von einer multifaktoriellen Ätiologie auszugehen [3, 27]. Da eine plausible Ätiologiekette zum gegenwärtigen Zeitpunkt fehlt, ist bis jetzt keine wirksame Präventionsstrategie umsetzbar, was als unbefriedigend zu beurteilen ist. Dies unterstreicht die Notwendigkeit weiterer Anstrengungen, um die genauen Ursachen für die Störung der Zahnhartsubstanzentwicklung zu identifizieren.

## Footnotes

1 https://generationr.nl/.

2 COPSAC: Copenhagen Prospective Studies on Asthma in Children (http://copsac.com/home/about/).

3 https://www.life.uni-leipzig.de/de/life_child/life_child.html.

4 GINIplus: The German Infant Study on the Influence of Nutrition Intervention plus Environmental and Genetic Influences on Allergy Development (https://www.ginistudie.de/).

5 LISA: Influence of Life-style Factors on Development of the Immune System and Allergies in East and West Germany plus Air Pollution and Genetics on Allergy Development (https://www.lisastudie.de/).
